# Viral activation and ecological restructuring characterize a microbiome axis of spaceflight-associated immune activation

**DOI:** 10.21203/rs.3.rs-2493867/v1

**Published:** 2023-10-10

**Authors:** Braden T. Tierney, JangKeun Kim, Eliah G. Overbey, Krista A. Ryon, Jonathan Foox, Maria Sierra, Chandrima Bhattacharya, Namita Damle, Deena Najjar, Jiwoon Park, Sebastian Garcia Medina, Nadia Houerbi, Cem Meydan, Jeremy Wain Hershberg, Jake Qiu, Ashley Kleinman, Gabe Al Ghalith, Matthew MacKay, Evan E Afshin, Raja Dhir, Joseph Borg, Christine Gatt, Nicholas Brereton, Ben Readhead, Semir Beyaz, Kasthuri J Venkateswaran, Kelly Blease, Juan Moreno, Andrew Boddicker, Junhua Zhao, Bryan Lajoie, Ryan T. Scott, Andrew Altomare, Semyon Kruglyak, Shawn Levy, George Church, Christopher E. Mason

**Affiliations:** 1 Department of Physiology and Biophysics, Weill Cornell Medicine, New York, NY, USA.; 2 The HRH Prince Alwaleed Bin Talal Bin Abdulaziz Alsaud Institute for Computational Biomedicine, Weill Cornell Medicine, New York, NY, USA; 3 Tri-Institutional Biology and Medicine program, Weill Cornell Medicine, New York, NY, USA; 4 Seed Health, Inc, Venice, CA, USA; 5 Swiss Institute of Allergy and Asthma Research (SIAF), University of Zurich, Davos, Switzerland; 6 Department of Applied Biomedical Science, Faculty of Health Sciences, University of Malta, Msida, MSD2090, Malta; 7 School of Biology and Environmental Science, University College Dublin, Dublin, Ireland; 8 ASU-Banner Neurodegenerative Disease Research Center, Arizona State University, Tempe, AZ, USA; 9 Cold Spring Harbor Laboratory, Cold Spring Harbor, NY, USA; 10 Jet Propulsion Laboratory, California Institute of Technology, Pasadena, CA , USA; 11 Element Biosciences, San Diego, CA, USA; 12 KBR; Space Biosciences Division, NASA Ames Research Center, Moffett Field, CA, USA; 13 Harvard Medical School and the Wyss Institute, Boston, MA, USA; 14 The WorldQuant Initiative for Quantitative Prediction, Weill Cornell Medicine, New York, NY, USA

## Abstract

Maintenance of astronaut health during spaceflight will require monitoring and potentially modulating their microbiomes, which play a role in some space-derived health disorders. However, documenting the response of microbiota to spaceflight has been difficult thus far due to mission constraints that lead to limited sampling. Here, we executed a six-month longitudinal study centered on a three-day flight to quantify the high-resolution microbiome response to spaceflight. Via paired metagenomics and metatranscriptomics alongside single immune profiling, we resolved a microbiome “architecture” of spaceflight characterized by time-dependent and taxonomically divergent microbiome alterations across 750 samples and ten body sites. We observed pan-phyletic viral activation and signs of persistent changes that, in the oral microbiome, yielded plaque-associated pathobionts with strong associations to immune cell gene expression. Further, we found enrichments of microbial genes associated with antibiotic production, toxin-antitoxin systems, and stress response enriched universally across the body sites. We also used strain-level tracking to measure the potential propagation of microbial species from the crew members to each other and the environment, identifying microbes that were prone to seed the capsule surface and move between the crew. Finally, we identified associations between microbiome and host immune cell shifts, proposing both a microbiome axis of immune changes during flight as well as the sources of some of those changes. In summary, these datasets and methods reveal connections between crew immunology, the microbiome, and their likely drivers and lay the groundwork for future microbiome studies of spaceflight.

## Introduction

The sources and impacts of spaceflight-associated microbiome shifts on astronaut health is an open yet important area of study. Microbes play manifold roles in human health, from acting as pathogens to symbionts; therefore, understanding the complex interplay between the space environment and host-microbiome composition is critical. This is especially true with the recent proliferation of commercial spaceflight missions and increased space tourism; individuals with increasingly diverse, microbiome-relevant medical histories will be traveling into space and to the Moon (e.g., dearMoon)^1^. In this new age, astronauts can be immunocompromised, cancer survivors, elderly, or have other health profiles that put them at greater risk of infection or other inclement outcomes, especially relative to prior NASA, ESA, JAXA, and ROSCOSMOS missions.^2^

Microbes are already associated with many spaceflight-specific health indications. In microgravity, many individuals experience gastrointestinal discomfort (i.e., constipation), which is heavily linked to gut microbiome composition^3–7^. The skin barrier is disrupted and often inflamed during and after flight, allowing potential invasion of pathobionts or otherwise inflammatory microorganisms^8–12^. Although the mechanisms are not entirely understood, the immune system experiences suppression during flight, leading to a “reactivation” of latent infections, such as herpes viruses. s^13–17^. As a result, identifying the sources and impacts of microbiome changes as a function of spaceflight will be essential for the development of microbiome-targeted, spaceflight-relevant diagnostics and therapeutics.

Microbial physiology, genetics, and community composition are also dramatically affected by the space environment, likely due to the stressors of microgravity and radiation^18–20^. These wide arrays of changes, taken together, radically alter the nature of microbial communities and, therefore, their cumulative impact on the host^21^. We recently documented the “ISS effect,” in which organisms on the International Space Station (ISS) exhibit increasing resistance to antibiotics over time, despite not having been exposed to them in the first place^22^. Many Biosafety Level 2 (BSL2) organisms, including *Haemophilus influenzae, Klebsiella pneumonia, Salmonella enterica, Shigella sonnei, and Staphylococcus aureus*, have been observed exhibiting ecological succession in the environment of the ISS, demonstrating the propensity of the space environment to select for specific community compositions and gene content.^19,23,24^. Finally, spaceflight alters biofilm formation capability in many bacteria; in some, like *Pseudomonas aeruginosa*, it increases the likelihood a superstructure will form, whereas in others, like *Proteus mirabilis*, it has the opposite effect^25,26^.

Indeed, early studies in aerospace medicine have indicated that the microbiome of humans and the built environment shift as a function of spaceflight^27^. These efforts, which have predominantly focused on the gut, have found convergence in astronaut microbiome signatures and shifts in the phylum ratios^27^. Studies of the oral cavity have identified decreases in *Streptococcus* and *Actinobacteriota* and increases in *Fusobacteriota* and *Proteobacteria* as a function of flight^28^.

However, there are many open questions regarding the microbiome architecture of spaceflight (see Glossary Supplementary Table 1), which we define as the totality of detectable, flight-associated, compositional, and expression shifts in the set of all bacteria, viruses, and microbial genes in the host and their surrounding environment. The proportion of organisms acquired from other crew members versus the environment remains unclear, the transience of microbiome changes post-flight remains opaque, and notably, the transcriptional activity of microbes as a response to flight is completely unexplored. These questions predominantly remain because prior studies have been hampered by 1) limited sample sizes, 2) a lack of longitudinal data, and 3) a focus on single sequencing modalities (i.e., amplicon sequencing). Commercial spaceflight, characterized by its high frequency and generally flexible parameters, offers a unique opportunity to address many of these limitations.

To further our understanding of microbiome community activity in spaceflight, we recently executed a longitudinal, multi-omic sampling study of the SpaceX Inspiration4 mission: the first all-civilian commercial flight to space. The Inspiration4 mission represented a unique opportunity to develop standards, as well as initial observations for measuring microbiome shifts during short-term spaceflight. Over a six-month window, the crew collected environmental (i.e., from the Dragon capsule), skin, nasal, and oral swabs at eight timepoints leading up to, during, and following a three-day mission in-orbit. We aimed to document, via metagenomics, metatranscriptomics, and host single cell sequencing, the bacterial and viral abundance and expression shifts and their relation to astronaut immune status. We focused on tracking expression and abundance shifts before flight, during flight, and after return to Earth. Specifically, we aimed to use metagenomics to gauge microbial abundance changes and metatranscriptomics to measure variation in microbial gene or species-marker-gene expression. We propose that our results yield a standardized approach for temporally monitoring microbial exposomic changes as a function of spaceflight and in total, characterize the microbiome architecture^29^ of biomedically relevant taxa that are potentially activated or repressed during short-term spaceflight.

## Results

### Quantifying the metagenomic architecture of short-term spaceflight

**Figure 1:**
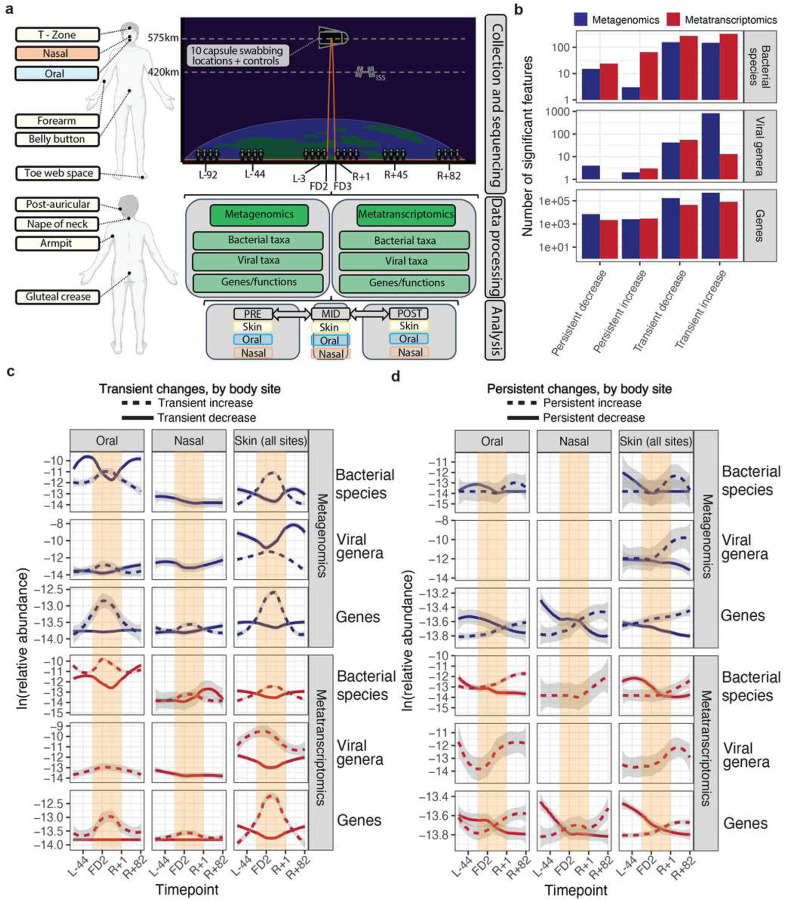
Overview of dataset and summary of alpha diversity. A) Collection and analytic approach. Body swabs were collected from ten different sites, comprising three microbial ecosystems (oral, nasal, skin) around the body at eight different timepoints surrounding launch. These are referred to as L-92, L-44, L-3, FD1, FD2, R+1, R+45, R+82, where “L-” refers to pre-launch, “FD” corresponds to flight day (i.e., mid-flight), “R” refers to recovery (i.e., post-flight). Following collection and paired metagenomic/metatranscriptomic sequencing, samples were processed to extract taxonomic (bacterial viral) and functional features to determine their changes relative to flight with a Microbiome Association Study (MAS). B) The total number of features (species or genes) found to be statistically associated with either pre- or post-flight timepoints across sequencing methods. Features are grouped by the categories laid out in the Methods regarding the nature of their changes relative to flight. C) The time trajectories of transiently increased/decreased significant findings across sequencing type, feature type, and body site (after filtering to remove low priority [i.e., weakly significant]) associations. Blank plots had either no significant findings or none that met the filtering criteria. D) Same as D, except viewing associations that were categorized as potentially persistent after flight.

The crew collected a microbiome dataset spanning eight timepoints: three before flight, three after flight, and two during flight. In total, we sequenced 385 metagenomic and 365 metatranscriptomic swabs comprising ten body sites representing the oral, nasal, and skin microbiomes (Fig 1A), plus eight stool samples (from two subjects before and after flight). Locations inside the Dragon Capsule were swabbed twice in flight and once prior (a separate Capsule was utilized for crew training). All the data from this sequencing effort have been stored in a database and made accessible in the NASA Open Science Data Repository.

(OSD-572, OSD-573)(Overbey et. al [under review].

To account for variation due to database and algorithmic bias, we used a diverse set of short-read alignment and *de novo* assembly approaches to estimate the microbial community taxonomic and functional composition of our dataset (Supplementary Figure 1, Supplementary Tables 2–6, *Methods*). We observed that many of the swabs collected, especially those from the skin sites, comprised low biomass microbial communities; there are many documented challenges in analyzing these data^30,31^. To filter environmental contamination and the kitome^32^ influencing our findings, we collected and sequenced negative controls of both (1) the water that sterile swabs were dipped in prior to use as well as (2) the ambient air around the sites of sample collection and processing for sequencing. These samples were used to remove potential contaminants (Supplementary Table 8). Unless otherwise specified, data presented in the main text are decontaminated and from Xtree aligned to the Genome-Taxonomy-Database (GTDB), Xtree aligned to the non-redundant set of complete GenBank viral genomes, and gene catalog relative abundances (see *Methods* for the rationale and benchmarking efforts).

To evaluate our taxonomic profiling approach, we first compared the top ten genus-level classifications by body site before and after decontamination for each classifier in metagenomic and metatranscriptomic data (Supplementary Figures 2–8). The dominant genera in each niche exhibited minimal change before and after decontamination. We observed general concordance among the various classification methods; for instance, the predominant skin genera consistently identified included Staphylococcus, Cutibacterium, and Corynebacterium. i. The oral microbiome included *Streptococcus*, *Rothia, and Fusobacterium*. Kraken2, which uses a database comprising both eukaryotic and prokaryotic organisms, identified fungi in the skin microbiome, as expected. The swabs from the Dragon capsule predominantly contained a diverse array of environmental microbes.

### Short-term spaceflight alters skin, oral, and nasal microbiome community ecology and transcriptional activity

The potential to observe dynamic ecological shifts was driven, in part, by a correlation analysis that identified potential transient and sustained changes in bacterial community composition (Supplementary Figure 10). As a result, we then queried if short-term spaceflight altered overall bacterial and viral community composition and expression consistently across the astronauts. Via a linear mixed effect (LME) modeling approach, we executed a Microbiome-Association-Study (MAS), computing associations for each taxonomic rank and classifier between flight and the abundance of 1) bacteria species, 2) viral genera and non-redundant proteins. We grouped False Discovery Rate (FDR) significant (q-value < 0.05) features into four categories: transiently increased in-flight, transiently decreased in-flight, persistently increased in/after flight, and persistently decreased in/after flight (Supplementary Table 9). We additionally fit generalized linear models (GLMs) alongside LMEs and identified the two approaches to be generally concordant (Supplementary Figure 11).

In total, we observed a mostly transient restructuring of the oral, nasal, and skin microbiomes as a function of flight (Fig 1B–C). Across all ten sites swabbed and regressed, over 821,337 associations were statistically significant and grouped into one of the four categories of interest. These comprised 314,701 distinct microbial features: 792 were viral, 767 were bacterial, and the remaining were genes) The majority (73.5%) of significant and categorized features were transiently increased in abundance. 24.6% were transiently depleted during flight. 0.6% and 1.1% of features appeared to continually increase or decrease (respectively) following the crew’s return to Earth. The limited persistence of changes indicates that, while microbial communities may restructure in space, the relative abundance of altered organisms, as well as their gene expression, generally reset upon returning to Earth.

**Figure 2:**
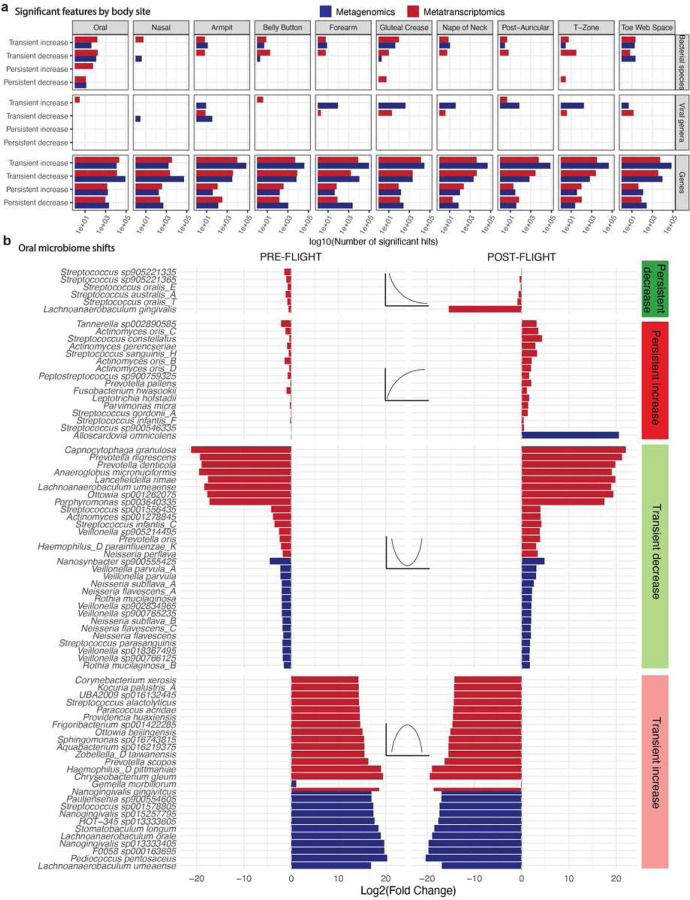
Site-specific changes and the oral microbiome architecture of spaceflight. A) Significant features by specific swabbing sites. B) The strongest associations between bacteria and flight for the oral microbiome. X-axes are average L2FC of all pre-flight or post-flight timepoints compared to the average mid-flight abundances for a given taxon.Columns correspond to different association categories that are described visually by the example line plots on top of each one. Dotted, gray, horizontal lines demarcate an L2FC of zero. Plotted taxa were selected by ranking significant features in each category by L2FC and showing up to 10 at once.

Different body sites displayed distinct time trends that varied depending on molecular type (gene expression vs. relative abundance) and domain of life. Time-dependent shifts were apparent in all body sites; average increases in relative abundance and gene expression tended to be greater than decreases (Fig 1C). Temporal trends were most striking for gene-level changes, which were identified across each body site. The oral microbiome also displayed a noticeable restructuring of both relative abundance and bacterial gene expression; 161 bacterial and viral taxonomies were transiently increased, 173 were transiently decreased, 62 were persistently increased, and 12 were persistently decreased (Fig 2A). Alternatively, the skin microbiome demonstrated almost no persistent changes and a higher proportion of relative abundance )but not necessarily gene expression) shifts, with 933 transiently increased (metagenomic) taxa across all eight skin sites. The number and direction of altered microbiome features were generally consistent across classification methods (Supplementary Figure 12), and most taxonomic associations were unique to individual body sites (Supplementary Fig 13).

### Skin and oral bacterial alterations are predominantly compositional in the former and metatranscriptomic in the latter

We next interrogated the specific taxonomic nature of bacterial shifts during spaceflight. Transient changes tended to have a larger log2(fold changes) [L2FC] of relative abundance or transcriptional activity than persistent ones, perhaps because even more lingering effects of flight tended towards returning to baseline by later timepoints. We also noted that the organisms with the strongest effects were different across biological modalities; in other words, an increase in gene expression did not necessarily imply the existence of a similar increase in the abundance of DNA ascribed to a given species. This discordance was apparent in the oral microbiome (Fig 2B), for example, where there was almost no overlap between the organisms that altered in terms of relative abundance and those that altered in terms of gene expression.

Overall, the oral microbiome demonstrated flight-dependent variation in the metatranscriptomic expression of bacteria associated with dental decay and biofilm formation (Fig 2B). Various members of *Fusobacteriota*, a progenitor to gum and tooth disease previously reported as spaceflight-associated, demonstrated an increase either in or after spaceflight^33^. These included *Fusobacterium hwasookii, Fusobacterium nucleatum* (Supplementary Table 9), and *Leptotrichia hofstadii*. Other oral biofilm species known to aggregate synergistically with *Fusobacterium* species in the mouth were also enriched in and after flight; these included *Streptococcus gordonii A*, multiple *Campylobacter* species, and *Actinomyces oris* species^34^. There was a persistent loss in the expression of *Streptococcus oralis spp*. and *Lachnoanaerobaculum gingivalis*, and a transient decrease in *Veillonella* spp. *Alloscardovia omnicolens* was the only organism with a strong, persistent increase in metagenomic DNA content. We compared the MetaPhlAn4 associations to those identified in GTDB and found similar results, especially regarding the overall enrichment of *Fusobacterium* sp., in flight.

**Figure 3:**
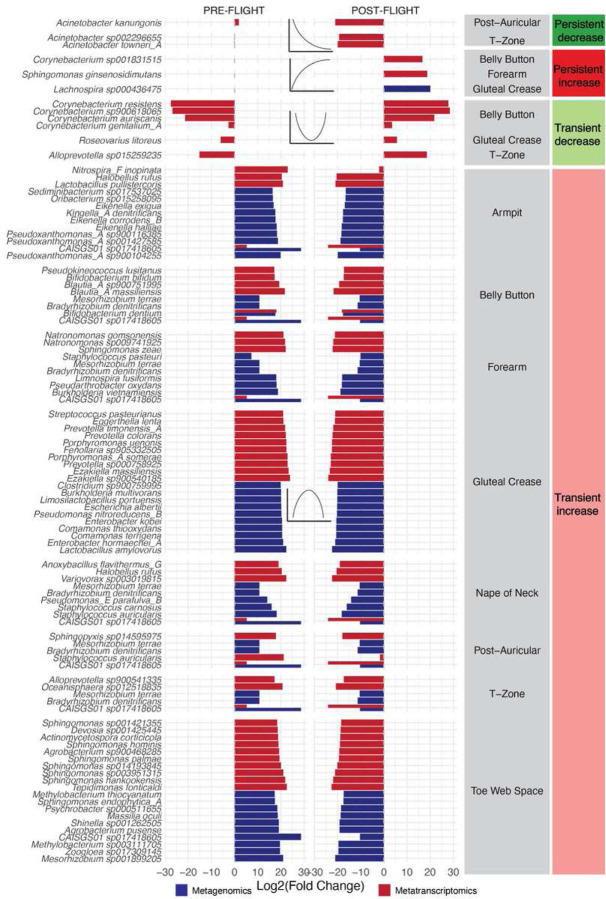
Strong changes to the skin microbiome during spaceflight. The strongest associations between bacteria and flight for the skin microbiome. X-axes are average L2FC of all pre or post flight timepoints compared to the average mid-flight abundances for a given taxon. Columns correspond to different association categories that are described visually by the example line plots on top of each one. Dotted, gray, horizontal lines demarcate an L2FC of zero. Plotted taxa were selected by ranking significant features in each category by L2FC and showing up to 10 at once.

Many of the strongest bacterial skin microbiome alterations (Fig 3) were predominantly metagenomic, as opposed to metatranscriptomic. We hypothesized that this may indicate the acquisition of new but non-transcriptionally active species from the surrounding environment. For example, persistent increases were mostly in the metagenomic content of various gut microbes (e.g., *Bacteroides*, *Parabacteroides, Blautia, Enterocloster*); this may result from altered hygiene habits during flight.

As with the oral microbiome, there was little concordance between metagenomic and metatranscriptomic changes. On the other hand, *Corynebacterium* species (common skin commensals) experienced metatranscriptomic, temporary depletion in-flight, and *Acinetobacter* spp. demonstrated a persistent depletion. These “typical” skin microbes (e.g., *Corynbacterium*, *Staphylococcus*, *Variovorax, Acinetobacter*) underwent changes in metatranscriptomic activity, whereas organisms not universally found on the human skin (*e.g., Mesorhizobium spp., Prevotella spp*.) tended to experience metagenomic shifts, again indicating the potential acquisition of niche-atypical, non-transcriptionally active organisms from the environment.

### Viral activation as a function of flight and host

**Figure 4:**
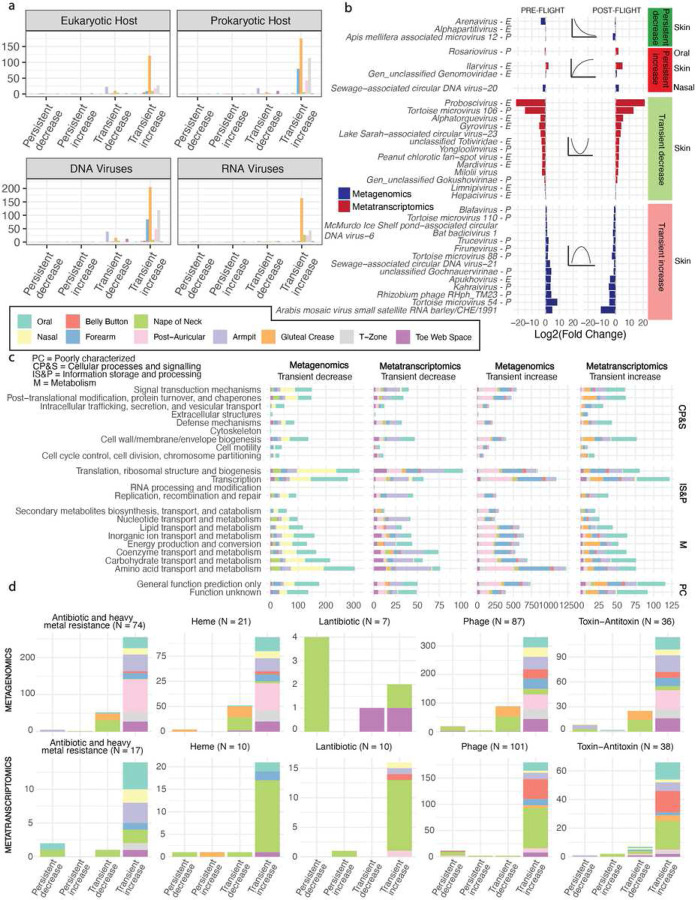
The viral and functional response of the microbiome to spaceflight A-B) Host and molecular type of viruses associated with flight, by category. B) The strongest associations between viruses and flight for the skin and oral microbiomes. X-axes are average L2FC of all pre-flight or post-flight timepoints compared to the average mid-flight abundances for a given taxon. Columns correspond to different association categories that are described visually by the example line plots on top of each one. Dotted, gray, horizontal lines demarcate an L2FC of zero. Plotted taxa were selected by ranking significant features in each category by L2FC and showing up to 10 at once. Viral genera are labeled “E” for targeting a eukaryotic host and “P” for targeting a prokaryote. If no definite host is known, no label was assigned. C) COG categories of all genes associated with flight. D) Groups of specific protein products that were associated with flight. The legend in the black box is relevant for all figures where those colors appear.

The landscape of viral activation and depletion covered both prokaryotic- and eukaryotic-targeting viral genera (Fig 4A). That said, the majority of detectable viral activity comprised phages in the skin microbiome (i.e., DNA viruses targeting prokaryotic hosts), and it was in large part concentrated in the gluteal crease. Most viral activity was transiently increased; in other words, even more dramatically than in the bacterial data, relatively speaking, viral abundances reset to baseline almost immediately after flight (Fig 4B).

Phylogenetically, viral activity appeared to be altered across diverse lineages (Supplementary Table 9, Fig 4B). For example, *Uroviricota*, *Cressdnaviricota*, and *Phixviricota* shifted across the oral, skin, and nasal microbiomes. However, phyla containing biomedically relevant, potential human pathogens increased, including *Kitrinoviricota*, *Artverviricota*, *Nucleocytoviricota*, and *Duplornaviricota*. A diverse set of genera – targeting both Eukaryotes and Prokaryotes – responses to flight (Fig 4B). The only persistently increased genera were *Rosariovirus, Ilarvirus*, and an unclassified *Genomoviridae*. Increased viral genera were mostly in the skin microbiome, and they almost entirely targeted prokaryotes. The decreased genera targeted mostly eukaryotic hosts and were detected via metatranscriptomics. These results indicate that viral activation is not a human-specific effect and occurs across all domains of life.

We compared these results at additional taxonomic ranks and with other taxonomic classifiers. For example, to discern higher specificity of the viral changes, we additionally fit species-level virus associations. While species-level viral taxonomic classification can be difficult due to high read misalignments (Supplementary Figure 14), we wanted to determine whether we could observe a higher-resolution picture of viral activation due to spaceflight, as this effect is known to be space-associated (as opposed to bacterial skin to skin transmission, which could be a result of sharing tight quarters and not a space-specific effect). The results we identified were in-line with the genus level but provided more detail. For example, we found transient increases in *Streptococcus* phages in the oral microbiome, potentially indicating a viral component to the substantial *Streptococcus*-associated ecological restructuring (as indicated in Fig 2B). An additional, more conservative approach for viral taxonomic classification (Phanta) further identified shifts in *Propionibacterium* and *Staphylococcus* phages in the skin microbiota (as well as an overall nasal microbiome increase in *Pisuviricota*, which contains many human pathogens).

### Towards a core functional microbial landscape of spaceflight

We next took a gene-level, taxonomy-agnostic approach to analyze the microbiome architecture of spaceflight. Both microbes and viruses rely on proteins for their functions; we theorized that spaceflight might induce consistent protein-level reactions across the functional units of the domains of life. We, therefore, aimed to characterize the consistency with which protein abundances changed across time and body site across 3.6 million non-redundant genes.

First, we explored the broad functions of the genes that fell into either the transiently increased or transiently decreased categories, once again observing body-site specific effects in-line with the taxonomic results (Fig 4C). The increases in DNA content on the skin, as well as decreases in nasal microbiome content, were immediately apparent (Fig 4C, third and first columns, respectively). The oral microbiome and gluteal crease underwent large metatranscriptomic increases. The category with the most genes – that exhibited the greatest fluctuation in gene number, both increasing and decreasing – was amino acid transport and metabolism. In the exposed areas of the skin microbiome, like the forearm, the genes that were changed in this category mostly came from metagenomic data. In less exposed body sites (i.e., oral, gluteal crease), the activity in this category was primarily metatranscriptomic. This may indicate the dramatic degree to which microbial nutrient needs change in-flight, likely from a combination of features, ranging from environmental strain transfer, competition, and host dietary changes.

The oral, nasal, and skin microbiomes demonstrated consistency in the functions that were altered during flight, especially in the metagenomic data. We observed five different categories of proteins of interest enriched among increased features: antibiotic and heavy metal resistance, heme binding/export, lantibiotic-associated proteins, phage-associated proteins, and toxin-antitoxin systems (Fig 4D, Supplementary Fig 15, Supplementary Table 9). Lantibiotic biosynthesis (Fig 4D, third column) again displayed a discordance between sequencing types; it was decreased in the metagenomic data but increased in metatranscriptomics. Heme-associated function expression increased in the oral microbiome, however, the number of genes detected metagenomically increased across all body sites. Phage proteins, toxin-antitoxin systems, and antibiotic/heavy metal pathways increased noticeably across host niches. We specifically observed an increase in the RelB toxin-antitoxin systems, most notably through metatranscriptomics. This finding was particularly interesting, as we and others have identified it as space-associated^22,35^.

### Strain-level tracking of microbial transfer between the capsule and astronauts

**Figure 5:**
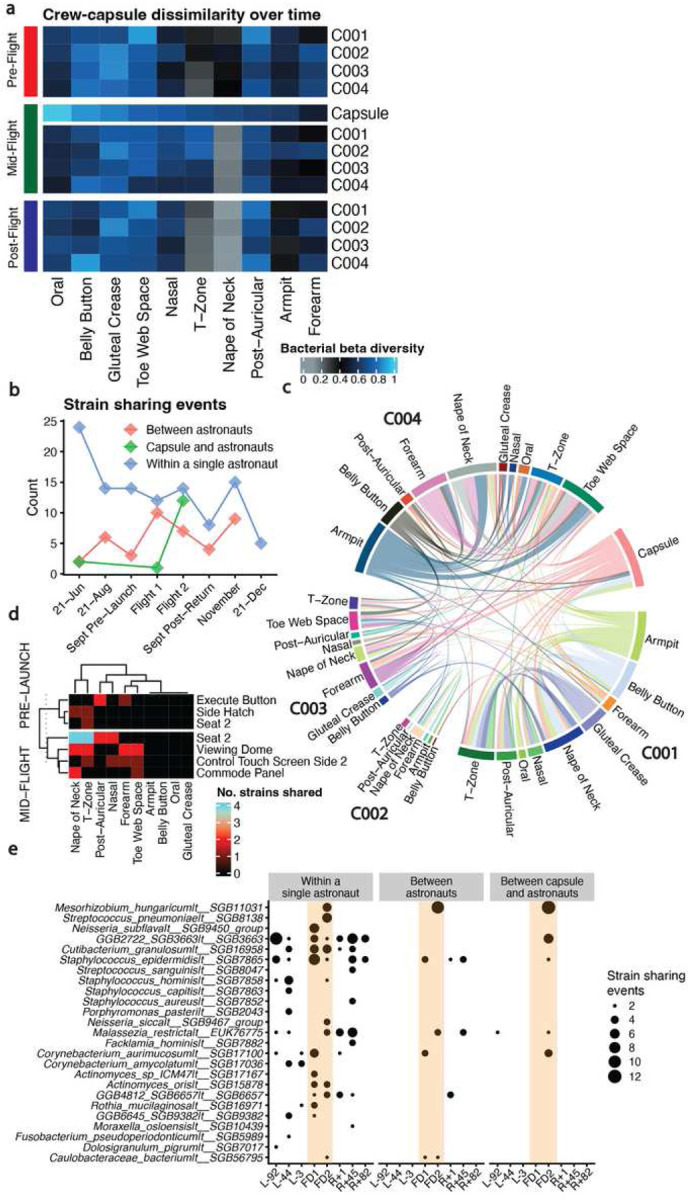
Microbial propagation through the Dragon Capsule and the crew. A) Beta diversities for bacterial metagenomics. Heatmap color corresponds to average beta diversity, with black being the midpoint (0.5), blue being totally dissimilar (1.0) and gray being highly similar (0.0). Columns are hierarchically clustered considering all rows. The interpretation for a single cell is, for the crew member annotated on the right-hand side, that body site’s dissimilarity to all other cells in that column (so the Capsule and all other crew samples from the same site). B) The number of strain-sharing events across time, where an event is defined as the detection of the same strain between two different swabbing locations. C) Strain sharing events between the crew and the capsule during the mid-flight timepoints. D) Capsule locations where strain sharing was identified in the training capsule and during flight. E) Organisms with at least two strain sharing events detected within a given timepoint.

We observed that, on average, bacterial beta diversity appeared to decrease after flight (Fig 5A). When ranking sites by similarity to the capsule mid-flight (Fig 5A, from left to right), the beta diversity correlated with the degree of environmental exposure for a given sampling site. For example, the oral microbiome remained highly dissimilar from the capsule and other sites, whereas the forearm became much more similar to the walls of the Dragon capsule and other crew members.

Further, our MAS indicated that, during flight, the composition of the astronaut’s microbiota changed, most notably in the skin niche, though the sources of these alterations were unclear. We hypothesized that these shifts in community composition and the overall increase in microbiome similarity could be a result simply of individuals cohabitating in a tight space; however, a change in gene expression in the oral microbiome (where strain exchange is possibly less likely), could derive from other ecological or other exposure changes like diet or immune alterations.

We aimed to determine if strain-tracking and individual microbiome dissimilarity could identify microbial transit between individuals and the environment, providing a potential explanation for a portion of our observed results. Specifically, we queried whether host microbiomes converged in similarity during and after flight and whether microbial exchange occurred within individuals, between individuals, or both within individuals and the capsule. We utilized recently-published methods^36^, using MetaPhlAn4 and StrainPhlAn, to determine if strain-level markers could discern the directionality of microbial exchange across environments.

Overall (Fig 5B), we found that individuals appeared to acquire strains from the capsule by the second mid-flight sampling point (day 3). During the L-92 timepoint, there was minimal transfer between the training capsule and the astronauts. Transfer within an individual (i.e.,single person’s body) remained relatively consistent across time. The majority of strain sharing occurred between the skin and the capsule swabs.

Considering only the in-flight timepoints (Fig 5C), we again noticed that most strain sharing occurred between sites on the same individual, with limited exchange between astronauts. Points on the capsule with high crew contact were a source of new skin diversity (Fig 5D, the seat, viewing dome, commode panel, control touch screen). Finally, the StrainPhlAn strains, like *Mesorhizobium_hungaricum|t__SGB11031* identified as present in multiple locations mid-flight (Fig 5E) were similar, in part, to those GTDB species identified as increased metagenomically (but not transcriptionally) across exposed skin sites (Fig 3). Notably, most of these shared strains between individuals were present after flight, as opposed to before.

### Spaceflight-associated microbiome shifts are correlated with immune cell gene expression

**Figure 6:**
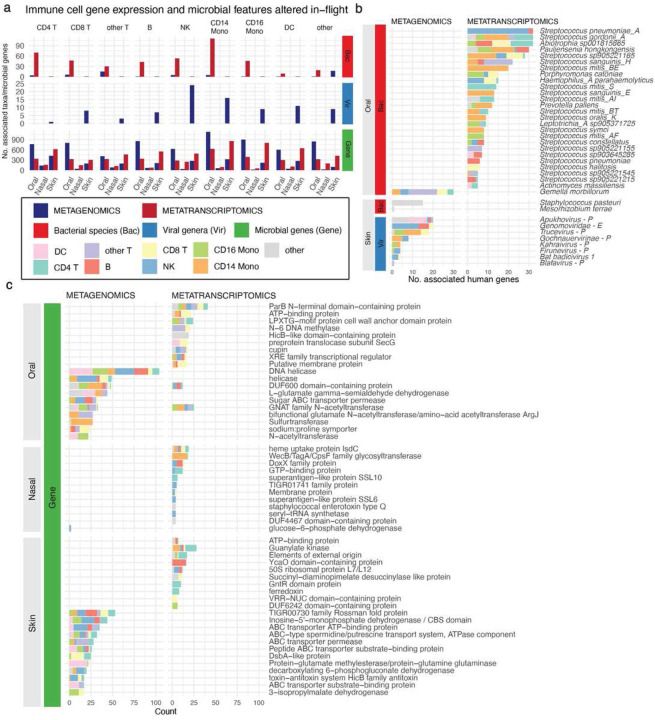
The landscape of potential immune-microbiome associations related to flight. A) The total number of microbial features, by type, associated with different immune cell subtypes for those that were long-term increased after flight (left panel) and decreased (right panel). B) The flight-associated (increased in abundance or expression) bacteria and viruses that were associated with the greatest number of host genes. Viral genera are labeled “E” for targeting a eukaryotic host and “P” for targeting a prokaryote. If no definite host is known, no label was assigned. C) The flight-associated microbial genes that were associated with the greatest number of host genes. We sorted for genes within each body site and selected the top 15 with the greatest number of human gene associations. The legend in the black box is relevant for all figures where those colors appear.

Having mapped the architecture of microbiome changes surrounding spaceflight and identified the source of some of those changes, we next searched for indications of a link between microbiome ecology and the host immune system. To do so, we integrated the observations from our MAS with host immune, single-cell data. Via averaging across single cell sequencing information, we estimated the gene expression of nine host immune cell subpopulations. We computed differentially expressed genes within cell types post-flight (Overbey et al. [in review], Kim et al., *Nature*. *In review*. ID: 2023-02-01822 ])(Fig 6). We used lasso regression to identify candidate relationships between flight-associated, increased microbial features and immune cell subpopulation gene expression (Supplementary Table 10), with the hypothesis that sustained changes to the microbiome would correlate to immune perturbations in the host.

We observed many putative relationships between host immune cell expression, body site, and microbial features (Fig 6A). Bacterial species – in the oral microbiome, specifically – had many metatranscriptomic associations across all cell types. In terms of relative abundance (i.e., metagenomics), oral microbes were associated with CD4 T cells, CD8 T cells, and CD16 monocytes, which are known for innate immune response against pathogens^37,38^. Skin bacteria had very few associations with immune cells (compared to oral) in both metagenomics and metatranscriptomics. The overall lack of bacterial metagenomic signal in the skin was interesting, as it indicated that strains acquired during flight that displayed altered relative abundance but limited transcriptional changes did not correlate to measurable host immune response. In other words, there was limited evidence that strain-sharing drove an altered immune state in humans.

There was a limited link in our data between viruses and immune cell expression. This was unsurprising, given that most of the altered viruses we were able to detect did not target human cells. Natural killer cells, CD14 monocytes, dendritic cells, and CD16 monocytes had the most viral associations. These associations were predominantly in the skin microbiome.

By cell type, we documented the most strongly associated genes with microbial features (Supplementary Table 10). For bacteria, gene functions were annotated with, for example, long non-coding RNAs (across all cell types), immunoglobulin genes (CD14 monocytes), and interferon regulatory factors. We additionally uncovered associations with specific immune modulatory genes such as CXCL10, XCL1, CXCL8 (immune cell migration), NLRC5, HLA genes, CD1C (antigen presentation/co-stimulation), SLC2A9 (immune cell metabolism), IRF1, NR4A3, STAT1 (transcription factors that specify immune cell states) that increased across multiple immune cell types (B cells, CD4 T-cells, CD8 T- cells, CD14 monocytes, DCs, Natural Killer (NK) cells).

Next, we examined a subset of microorganisms with expression and abundance changes that correlated to host genes across multiple cell types (Fig 6B). A small group of metagenomically-detected viruses were associated with many different immune genes; one genus (*Genomoviridae*) targets fungi and was correlated to a relatively large number (13) genes in natural killer cells. The presence of this virus on the skin makes additional sense given that fungi are known skin symbionts. The other associated viruses had unclassified hosts or targeted bacteria.

In the oral microbiome, pathobiont gene expression was associated with immune cell gene expression. *Streptococcus pneomoniae A* had the largest number of genes associated with it; 30/32 genes were found in natural killer cells. *Streptoccocus gordonii A*, which was persistently increased after flight was associated with many different immune cell subtypes (N = 32 genes), including CD4 Y cells, CD13 monocytes, CD16 monocytes, and dendritic cells. The only oral bacterial relative abundance increase during or after flight that was associated with many immune cell subtypes was in *Gemella morbillorum*. The other oral microbes with the strongest oral associations included other medically relevant organisms, as well as some typical commensals: *Pauljensenia hongkongensis, Campylobacter_A concisus_R, Actinomyces massiliensis, Haemophilus_A parahaemolyticus*, *Leptotrichia_A sp905371725*, *Porphyromonas catoniae*, and many *Streptococcus spp*.

The microbial genes (Fig 6C) associated with the most human genes were detected by both shifts in relative abundance as well as expression. They spanned many different protein annotations, yet there were some commonalities among those that were correlated to many immune cell subpopulations. Most notably, these annotations – across both metagenomics and metatranscriptomics – included transcription factors, cell surface proteins, and transporters. Pertinent to our prior results (Fig 4), the top microbial gene in the nasal microbiome was a heme uptake protein.

### Discussion

In this study, which comprises the largest dataset of space-flight-associated microbiome data to date, we systematically queried the microbiome architecture of short-term spaceflight. Prior efforts, like the NASA twins study, have had difficulty identifying microbiome shifts due to small sample sizes and limited sequencing modalities^27^. Via comparing metagenomics and metatranscriptomics, we identified microbiome changes that indicate how, even over short periods of time, the effect of spaceflight can potentially impact astronaut microbiomes. We found bacterial taxa, viral taxa, and genes that were enriched or depleted during and after flight. Despite the mission only lasting three days, the oral, nasal, and skin microbiota of the host dramatically restructured their composition and expression. These alterations varied longitudinally, with some persisting and correlated to expression changes in host immune cells.

The sources of astronaut immune changes during flight are not well understood; however, we suggest a potential microbial axis as a contributing factor to this documented effect. We hypothesize our results may indicate how microbiome ecology associates could feasibly affect host immune function. First, we observed evidence of microbiome restructuring along the lines of potential interspecies interaction, stress response, and microbial energy source utilization shifts (Fig 5B–C, Supplementary Table 9). Pan-phyletic viral activation – and repression – were additionally noticeable (Fig 4). The oral microbiome – and other niches – underwent a metatranscriptomic “switch” (Fig 1C) between enriched and depleted expression signals in-flight. Changes appeared to derive from both bacteriophage activity and, for instance, downregulation and upregulation of different microbial species (like, *Streptococcus* [Fig 1C, Fig 2B]). Additionally, upon returning to Earth, astronauts experienced some persistent reorganization of community structure and function across their bodies. We identified that microbiome changes deriving from relative abundance changes (i.e., exchange of strains on the skin) are unlikely to be correlated to host immune response. Instead, microbiome alterations (i.e., gene expression shifts) deriving from sources other than cohabitation were more likely to be associated with host immune state (Fig 6).

Naturally, a microbial shift can affect the host immune system – or vice versa – without the initial cause being “space-specific” (i.e., due to microgravity of radiation). Strain sharing, for example, could be – and likely is – a function of humans sharing close quarters. Other changes, like periodontal pathogens, could stem from oral cleaning differing in space than on Earth. However, we hypothesize that at least some immune-associated microbiome alterations likely are due to exposure to the space environment and the immune alterations that occur as a function of flight. For example, astronauts have been documented as experiencing immune and viral activation^15^; typically, this effect is not attributed solely to cohabitation. Further, we see a clear difference between microbial cell acquisition in metagenomic data and the niche-native taxa that drove activity in the metatranscriptomic data. We claim it is unlikely strain sharing due to close quarters – or even variable sanitation in-flight – explains the entirety of the link between host immune response and the microbiome.

A large component of our findings centers on the discordance between microbial gene expression and microbial abundance; the former seems to have a larger relationship to space-associated and host immune shifts than the latter. Transcriptional changes dominated the oral microbiome, whereas exposed skin was dominated by metagenomic changes. This indicates a greater acquisition of foreign and transcriptionally inactive microbes between crew members and/or the environment. Most microbial exchange was between different sites within the same person or from within the built environment to individuals, as opposed to from person-to-person (Fig 5). However, both skin and oral changes did demonstrate strong correlations to changes in multiple immune cell types, indicating how microbiome shifts stemming from distinct underlying causes can mutually influence host health.

Future missions may also show the same core set of functional elements that were ostensibly species-independent and enriched in-flight. Some of the other conserved, increased functions across body sites have been reported in prior studies. For example, the RelB/E toxin-antitoxin systems enriched in *Acinetobacter pittii* on the ISS^22^. In the metatranscriptomic data, RelB-associated systems increased during flight. The increase of these and other defensive and antibiotic production metabolisms is of particular note, as it may form the basis of an “ISS effect” – where increases in bacterial antibiotic resistance occur, despite no exposure to antibiotics^22^.

A major limitation of our work is its descriptive nature, which arises from the overall study design. Despite having more samples than other astronaut microbiome studies, this effort still hosts a relatively small crew size (n = 4), and we cannot determine from these data alone if an outside effect on the immune system is altering their abundance or expression or if viral ecology may be driving these and similar changes. Given the nascence of the multi-omic space biomedicine (and the difficulty of sample collection), we were limited in this study to simply observing shifts in microbes and, from strain tracking and multi-omic data integration, inferring hypotheses regarding the overall nature of the mid-flight microbe-immune axis. Some of our identified associations may be individual or flight-specific.

As such, there are several opportunities to expand upon this work in future studies and missions. Analytically, our lasso-based approach for immune-microbe-interaction modeling immune changes does not inherently allow for statistical inference or account for inter-individual variation. Further, some of our samples had very low biomass, requiring PCR-amplification (18 cycles) for RNA-sequencing data, which can increase duplicate rates of sequences. For this reason, we attempted to take a conservative and systematic modeling approach to our effort. Specifically, 1) we implemented multiple algorithms and compared their concordance, 2) set coverage thresholds for bacterial and viral taxa to filter probable false positives, 3) used multiple, state-of-the-art taxonomic classifiers and compared our findings among all of them, and 4) implemented and compared both generalized linear models and mixed effect models, bearing in mind that the latter can face interpretability challenges with smaller sample sizes. We additionally used 76 negative controls to attempt to avert false positive signals, which can stem from contamination and the kitome. However, this approach is far from perfect and likely removes present organisms. Depending on their aim, future studies should alter collection methods to increase the amount of biomass collected sampling (e.g., using one swab for multiple skin sites) or examine relatively unbiased methods of amplification^40^.

Additional experiments and missions can further test a microbiome-derived theory of spaceflight-associated immune changes. In addition to stress-testing our findings and increasing sample sizes, future spaceflight studies should consider several enhancements. For instance, they should compare sequestered ground controls to discern differences between space-driven and proximity-driven immune shifts. Additionally, future efforts should design experiments that enable a deeper view into the causality of microbe immune associations rather than just noting their existence. Exploring some of these hypotheses through animal or organoid models could be valuable.

In total, spaceflight microbiome studies are hyperbolic extensions of unique kinds of human exposome research. They capture a group of effectively immunocompromised individuals who share a self-contained environment that does not undergo microbial exchange with the outside world. Since these studies are rare, the range of immune system dynamics is just beginning to be explored. Overall, we describe here data and methods to map the axes of host-microbe-environment interaction such that these observations and hypotheses can be tested in future studies. Indeed, the increased access to space guarantees more opportunities to study astronauts, their microbiomes, and their spacecraft while also motivating a strong health and medical impetus to plan for future missions.

## Methods

### Informed consent and IRB approval

All subjects were consented at an informed consent briefing (ICB) at SpaceX (Hawthorne, CA), and samples were collected and processed under the approval of the Institutional Review Board (IRB) at Weill Cornell Medicine, under Protocol 21–05023569. All crew members have consented for data and sample sharing.

### Sample collection, extraction, and sequencing

We sequenced analyzed samples from human skin, oral, and nasal environmental swabs before, during, and after a 3-day mission to space. This dataset comprised paired metagenomic and metatranscriptomic sequencing for each swab. A total of 750 samples were analyzed in this study by the four crew members of the Inspiration4 mission. They were taken from ten body sites (Fig 1A) across eight collection points (3 pre-launch, 2 mid-flight and 3 post-flight) between June of 2021 and December of 2021. They additionally collected twenty samples from multiple Dragon Capsules from ten different locations. A full description of the sample collection and sequencing methods are available in Overbey *et al*. (Collection of Biospecimens from the Inspiration4 Mission Establishes the Standard Omics Measures for Astronauts (SOMA) Initiative [in review, *Nature Methods*]) and Overbey *et al*. (The Space Omics and Medical Atlas (SOMA): A comprehensive data resource and biobank for astronauts [in review, *Nature Communications*]).

The crew were each provided sterile Isohelix Buccal Mini Swabs (Isohelix, #cat MS-03) and 1.0mL dual-barcoded screw-top tubes (Thermo Scientific, cat# 3741-WP1D-BR/1.0mL) prefilled with 400uL of DNA/RNA Shield storage preservative (Zymo Research, cat# R1100). Following sample collection, swabs were immediately transferred to the barcoded screw-top tubes and kept at room temperature for less than 4 days before being stored at 4C until processing.

DNA, RNA and proteins were isolated from each sample using the QIAGEN AllPrep DNA/RNA/Protein Kit (QIAGEN, cat# 47054) according to the manufacturer’s protocol, yet omitting steps one and two. In order to lyse biological material from each sample, 350uL of each sample was transferred to a QIAGEN PowerBead Tubes with 0.1mm glass beads and secured to a Vortex-Genie 2 using an adapter (cat# 1300-V1–24) before being homogenized for 10 minutes. 350uL of the subsequent lysate was then transferred to a spin-column before proceeding with the protocol. Concentration of the isolated DNA, RNA and protein for each sample were measured by fluorometric quantitation using the Qubit 4 Fluorometer (Thermo Fisher Scientific, cat# Q33238) and a corresponding assay kit. The Qubit 1Xds DNA HS Assay Kit was used for DNA concentration (cat# Q33231) and the RNA HS Assay Kit (cat# Q32855) was used for RNA concentration.

For shotgun metagenomic sequencing, library preparation for Illumina NGS platforms was performed using the Illumina DNA FLEX Library prep kit (cat# 20018705) with IDT for Illumina DNA/RNA US Indexes (cat# 20060059). Following library preparation, quality control was assessed using a BioAnalyzer 2100 (Agilent, cat# G2939BA) and the High Sensitivity DNA assay. All libraries were pooled and sequenced on a S4 flow cell of the Illumina NovaSeq 6000 Sequencing System with 2 × 150 bp paired-end reads.

For metatranscriptomic sequencing, library preparation and sequencing were performed at Discovery Life Sciences (Huntsville, Alabama). The extracted RNA went through an initial purification and cleanup with DNase digestion using the Zymo Research RNA Clean & Concentrator Magbead Kit (cat# R1082) per the manufacturer’s recommended protocol on the Beckman Coulter Biomek i5 liquid handler (cat# B87583). Following cleanup, rRNA reduction for RNA-seq library reactions were performed using New England Bioscience (NEB) NEBnext rRNA Depletion Kit (Human/Mouse/Rat) (cat# E6310X) and libraries were prepared using the NEB NEBnext Ultra II Directional RNA Library Prep Kit (cat# E7760X) with GSL 8.8 IDT Plate Set B indexes. Following library preparation, quality control was assessed using the Roche KAPA Library Quantification Kit (cat# KK4824). All libraries were pooled and sequenced on a S4 flow cell of the Illumina NovaSeq 6000 Sequencing System with 2 × 150 bp paired-end reads.

For fecal collection, all subjects are provided with DNA Genotek OMNIgene-GUT (OM-200) kits for gut microbiome DNA collection. Each subject was instructed to empty their bladder and collect a fecal sample free of urine and toilet water. From the fecal specimen, each subject used a sterile single-use spatula, provided by the OMNIgene-GUT kit, to collect the feces and deposit it into the OMIgene-GUT tube. Once deposited and sealed, the user was instructed to shake the sealed tube for 30 seconds in order to homogenize the sample and release the storage buffer. All samples from each timepoint were stored at room temperature for less than 3 days before storing at −80°C long-term. Fecal samples collected using the OMNIgene-GUT kit are stable at room temperature (15°C to 25°C) for up to 60 days.

DNA was isolated from each sample using the QIAGEN PowerFecal Pro DNA Kit (cat# 51804). OMNIgene-GUT tubes thawed on ice (4°C) and vortexed for 10 seconds before transferring 400uL of homogenized feces into the QIAGEN PowerBead Pro Tube with 0.1mm glass beads and secured to a Vortex-Genie 2 using an adapter (cat# 1300-V1–24) before being homogenized at maximum speed for 10 minutes. The remainder of the protocol was completed as instructed by the manufacturer. The concentration of the isolated DNA was measured by fluorometric quantitation using the Qubit 4 Fluorometer (Thermo Fisher Scientific, cat# Q33238), and the Qubit 1Xds DNA Broad Range Assay Kit was used for DNA concentration (cat# Q33265).

For shotgun metagenomic sequencing, library preparation for Illumina NGS platforms was performed using the Illumina DNA FLEX Library prep kit (cat# 20018705) with IDT for Illumina DNA/RNA US Indexes (cat# 20060059). Following library preparation, quality control was assessed using a BioAnalyzer 2100 (Agilent, cat# G2939BA) and the High Sensitivity DNA assay. All libraries were pooled and sequenced on the Illumina NextSeq 2000 Sequencing System with 2 × 150 bp paired-end reads.

### Sample quality control

All metagenomic and metatranscriptomic samples underwent the same quality control pipeline prior to downstream analysis. Software used was run with the default settings unless otherwise specified. The majority of our quality control pipeline makes use of bbtools (V38.92), starting with clumpify [parameters: optical=f, dupesubs=2,dedupe=t] to group reads, bbduk [parameters: qout=33 trd=t hdist=1 k=27 ktrim=“r” mink=8 overwrite=true trimq=10 qtrim=‘rl’ threads=10 minlength=51 maxns=−1 minbasefrequency=0.05 ecco=f] to remove adapter contamination, and tadpole [parameters: mode=correct, ecc=t, ecco=t] to remove sequencing error.^41^ Unmatching reads were removed using bbtool’s repair function. Alignment to the human genome with Bowtie2 (parameters: --very-sensitive-local) was done to remove potentially human-contaminating reads.^42^

### Metagenomic assembly, bacterial and viral binning, and bin abundance quantification

We assembled all samples with MetaSPAdes V3.14.3 (--assembler-only).^43^ Assembly quality was gauged using MetaQUAST V5.0.2.^44^ We binned contigs into bacterial Metagenome-Assembled-Genomes on a sample-by-sample basis using MetaBAT2 [parameters: –minContig 1500].^45^ Depth files were generated with MetaBAT2’s built-in “jgi_summarize_bam_contig_depths” function. Alignments used in the binning process were created with Bowtie2 V2.2.3 [parameters: —very-sensitive-local] and formatted them into index bamfiles with samtools V1.0.

Genome bin quality was checked using the “lineage” workflow of CheckM V1.2.^46^. Medium and high-quality bins were dereplicated using deRep V3.2.2 [parameters: -p 15 -comp 50 -pa 0.9 -sa 0.95 -nc 0.30 -cm larger]. The resulting database of non-redundant bins was formatted as an xtree database [parameters: xtree BUILD k 29 comp 2], and sample-by-sample alignments and relative abundances were completed with the same approach as before. Bins were assigned taxonomic annotations with GTDB-tK.^47^

### Identification and taxonomic annotation of assembled viral contigs

To identify putative viral contigs, we used CheckV V0.8.1.^48^ For downstream viral abundance quantification, we filtered for contigs annotated as medium quality, high quality, or complete. This contig database was dereplicated using BLAST and clustered at the 99% identity threshold as described above using, the established and published approaches (https://github.com/snayfach/MGV/tree/master/ani_cluster)^49^. The non-redundant viral contigs were formatted as an xtree database [parameters: xtree BUILD k 29 comp 0], and sample-by-sample alignments and relative abundances were computed with the same approach as before, the only difference between the coverage cutoff used to filter out viral genomes, which was lowered to 1% total and 0.05% unique due to the fact that those in question came directly from the samples analyzed.

We also aimed to assign taxonomy to putative viral contigs based on domain overlap with the GenBank reference database. We used a Hidden Markov Model (HMM) based approach (https://github.com/b-tierney/vironomy) to detect shared, single copy genetic features between query and reference genomes (from the pFam and TIGRFAM databases)^50,51^. Potential phyla were identified by screening the top five most similar reference genomes to those in the given query dataset.

### Gene catalog construction and functional annotation

We generated gene catalogs using an approach piloted in prior studies.^52–54^. Bakta V1.5.1 was used to call putative Open-Reading-Frames (ORFs).^55^ The annotations reported in this study (e.g., Fig 5) derive directly from Bakta. We clustered predicted and translated ORFs (at 90% requisite overlap and 90% identity) into homology-based sequence clusters using MMseqs2 V13.4511^56^ [parameters: –easy-cluster –min-seq-id 0.9 -c 0.9]. The resulting “non-redundant” gene catalog and its annotations was used in the functional analysis. We computed the abundance of the representative, consensus sequences selected by MMseqs2 by alignment of quality-controlled reads with Diamond V2.0.14.^57^ We computed the total number of hits and computed gene relative abundance by dividing the number of aligned reads to a given gene by its length and then the total number of aligned reads across all genes in a sample.

### Benchmarking short read viral taxonomic classification against the GenBank database

To identify viral taxonomic abundance via short read alignment, we mapped reads to a database of all complete, dereplicated (by BLAST at 99% sequence identity) GenBank viral genomes. We used the Xtree aligner for this method (see below), however given the difficulty of assigning taxonomic ranks to viral species based on alignment alone, we first benchmarked this process. We used Art(Huang et al. 2012) to generate synthetic viral communities at random abundances from 100 random viruses from the GenBank database. We then aligned (with Xtree) back to these genomes, filtered for 1% total coverage and/or 0.5% unique coverage, and compared expected read mapping vs. observed read mapping. We additionally computed True/False positive rates based on the proportion of taxa identified that were present in the mock community (True positive) versus those that were not (False positive) versus those that were present but not identified (False negative). Overall, we identified optimal classification at the genus-level, with >98% true positive rate (i.e., 98/100 taxa identified) and low false positive/negative rates (e.g., <10 taxa not present in the sample identified) (Supplementary Figure 14A-B). Species-level classification had higher false negative rates (generally arising from multi-mapping reads to highly similar species) and a 60–70% true positive rate. Genus level classification also yielded a nearly perfect correlation (>0.99, on average) between expected and observed read mappings (Supplementary Figure 14C). As a result, while we report analyses for every taxonomic rank in the supplement, in the main text we describe only genus-level viral analysis.

### Short-read taxonomic classification via alignment

In total, we used and compared seven different short read mapping methods (MetaPhlAn4/StrainPhlAn, Xtree, Kraken2/Bracken run with four different settings, Phanta), which together utilize five different databases that span bacterial, viral, and fungal life. Additionally, we identified and computed the relative abundance of non-redundant genes as well as bacterial and viral Metagenome-Assembled-Genomes (Supplementary Table 7). Subsequent downstream regression analyses were run on each resultant abundance table at each taxonomic rank.

Unless otherwise stated, for the figures involving taxonomic data used in the main text of the manuscript, we used the XTree (https://github.com/GabeAl/UTree) [parameters: –redistribute]. XTree is a recent update to Utree^58^, containing an optimized alignment approach and increased ease of use. In brief, it is a k-mer based aligner (akin to Kraken2^59^ but faster and designed for larger databases) that uses capitalist read redistribution^60^ in order to pick the highest-likelihood mapping between a read and a given reference based on the overall support of all reads in a sample for said reference. It reports the total coverage of a given query genome, as well as total unique coverage, which refers to coverage of regions found in only one genome of an entire genome database.

For bacterial alignments, we generated an Xtree k-mer database [parameters: BUILD k 29 comp 0] from the Genome Taxonomy Database representative species dataset (Release 207) and aligned both metagenomic and metatranscriptomic samples. We filtered bacterial and genomes for those that had at least 5% coverage and/or 2.5% unique coverage. Relative abundance was calculated by dividing the total reads assigned to a given genome by the total number of reads assigned to all genomes in a given sample. We additionally ran MetaPhlAn4^61^ (default settings) as an alternative approach to bacterial taxonomic classification.

For viral GenBank alignments, we generated an Xtree database [parameters: BUILD k 17 comp 0] from all complete GenBank viral genomes. We first de-replicated these sequences with BLAST 99% identity threshold via published approaches (https://github.com/snayfach/MGV/tree/master/ani_cluster).^49,62^ We filtered for genomes with either 1%/0.5% total/unique coverage. Relative abundance was calculated identically as with the bacterial samples. We additionally ran Phanta (default settings) as an alternative to this approach for viral classification^63^.

As another set of methods for measuring taxonomic sample composition, we used Kraken2 and bracken, both with the default settings, to call taxa and quantify their abundances, respectively.^59,64^ We used the default kraken2 reference databases, which includes all NCBI listed taxa (bacteria, fungal, and viral genomes) in RefSeq, as of September 2022. We ran Kraken2 with four different settings: default (confidence = 0) and unmasked reads, confidence = 0 and masked reads, confidence = 0.2 and unmasked reads, and confidence = 0.2 and masked reads. In the cases where we masked reads prior to alignment (to filter repeats and determine if fungal and other eukaryotic alignments were likely false positives), we used bbmask running the default settings.

Finally, we computed beta diversity (Bray-Curtis) metrics for taxonomic abundances using the vegan package in R.^65^

### Sample decontamination with negative controls

Following taxonomic classification and identification of *de novo* assembled microbial genes, we removed potential contaminants from samples by comparison to our negative controls (detailed in Supplementary Table 8). We ran the same classification approaches for each negative control sample as described in the above paragraphs in this section. This yielded, for every taxonomy classification approach and accompanying database, a dataframe of negative controls alongside a companion dataframe of experimental data. On each of these dataframe pairs, we then used the isContaminant function (parameters: method=“prevalence”, threshold = 0.5) of the decontam package^66^ to mutually high prevalence taxa between the negative controls and experimental samples. The guidance for implementation of the decontam package, including the parameter used, was derived from the following R vignette: https://benjjneb.github.io/decontam/vignettes/decontam_intro.html. Note that we used both metagenomic and metatranscriptomic negative control samples to decontaminate all data, regardless of if that data was itself metagenomic or metatranscriptomic. This decision was made to increase the overall conservatism of our approach..

### Metagenomic-Association-Study on bacteria, viruses, and genes

Four mixed-model specifications were used for identifying microbial feature relationships with flight. Time is a variable encoded with three levels corresponding to the time of sampling relative to flight: PRE-FLIGHT, MID-FLIGHT, and POST-FLIGHT. The reference group was the MID-FLIGHT timepoint, indicating that any regression coefficients had to be interpreted relative to flight (i.e., a negative coefficient on the pre-launch timepoint implies that a feature was increased in-flight). We fit these models for all genes, viruses, and bacteria identified in our dataset by assembly, XTree (GTDB/GenBank), MetaPhlAn4, Kraken2 (all four algorithmic specifications), Phanta, and gene catalog construction. Each variable encoding a body site is binary encoding if a sample did or did not come from a particular region.

To search for features that were changed across the entire body, we fit overall associations, oral associations, skin associations, and nasal associations.:

1.
$ln(microbial\_feature\_abundance\;+\;minval\;)\sim \beta_{0}+\beta_{1}Time\;+(1|Crew.ID\;)+\epsilon_{i}$

Whereas, for associations with oral changes, we used:

-
$ln(microbial\_feature\_abundance\;\;+\;minval\;)\sim \beta_{0}+\beta_{1}Time\;*Oral+(1|Crew.ID\;)+\epsilon_{i}$

Whereas, for associations with nasal changes, we used:

-
$ln(microbial\_feature\_abundance\;+\;minval\;)\sim \beta_{0}+\beta_{1}\;Time\;*\;Nasal\;+(1|Crew.ID\;)+\epsilon_{i}$

For identifying associations with skin swabs, we fit the following model:

-
$\begin{array}{l} ln(microbial\_feature\_abundance\;+\;minval\;)\sim \beta_{0}+\beta_{1}\;Time\;*\;Armpit\;+\\ \beta_{2}Time\;*\;ToeWeb\;+\beta_{3}\;Time\;*\;NapeOfNeck\;+\beta_{4}\;Time\;*\;Postauricular\;+\\ \beta_{5}Time\;*\;Forehead\;+\beta_{6}\;Time\;*\;BellyButton\;+\beta_{7}\;Time\;*\;GlutealCrease\;+\\ \beta_{8}Time\;*\;TZone\;+(1|Crew.ID\;)+\epsilon_{i} \end{array}$

Note that in this final equation (4), the reference groups are samples deriving from the nasal and oral microbiomes; this means that highlighted taxa will be those associated with time and skin sites as compared to the oral and nasal sites. We additionally fit these same model specifications without the random effect and compared the results in Supplementary Figure 11.

We used the lme4^67^ package to compute associations between microbial features (i.e., taxa or genes) abundance and time as a function of spaceflight and bodysite. For all data types, we aimed to remove potential contamination prior to running any associations. We estimated p-values on all models with the LmerTest packages using the default settings.^67,68^ We adjusted for false positives by Benjaini-Hochberg adjustment and used a q-value cutoff point of 0.05 to gauge significance.

### Identifying and plotting time-dependent trends in microbial features

We grouped microbial features associated with flight into six different categories. These were determined due to the fact that our model contained a categorical variable encoding a sample’s timing relative to flight: whether it was taken before, during, or afterwards. Since the modeling reference group was “MID-FLIGHT,” meaning that the interpretation of any coefficients would be directionally oriented relative to mid-flight microbial feature abundances. As a result, we were able to categorize features based on the jointly considered direction of association and significance for the “PRE-FLIGHT” and “POST-FLIGHT” levels of this variable. The below listed categories are all included in the association summaries provided in Supplementary Table 3.

1. Transient increase in-flight – negative coefficient on the PRE-FLIGHT variable level, negative coefficient on the POST-FLIGHT variable, statistically significant for both
2. Transient increase in-flight (low priority) – negative coefficient on the PRE-FLIGHT variable level, negative coefficient on the POST-FLIGHT variable, statistically significant for at least one of the two
3. Transient decrease in-flight – positive coefficient on the PRE-FLIGHT variable level, positive coefficient on the POST-FLIGHT variable level, statistically significant for both
4. Transient decrease in-flight (low priority) – positive coefficient on the PRE-FLIGHT variable level, positive coefficient on the POST-FLIGHT variable level, statistically significant for at least one of the two
5. Potential persistent increase – negative coefficient on the PRE-FLIGHT variable level, positive coefficient on the POST-FLIGHT variable level, statistically significant for at least one of the two
6. Potential persistent decrease – positive coefficient on the PRE-FLIGHT variable level, negative coefficient on the POST-FLIGHT variable level, statistically significant for at least one of the two

We used these groups to surmise the time trends reported in Figures 1, 2, 3, 4, and Supplementary Figures 15–17. It would be intractable to visualize every association of interest, so we prioritized within each category based on the absolute value of beta-coefficients and adjusted p-values. In Figure 1C, we removed the “low priority” categories (two and four above) and only looked at the top 100 most increased and decreased significant genes, by group, relative to flight. We did so to make fitting splines feasible (especially in the case of genes, which had so many associations), and filter out additional noise due to low association-size findings.

We took a similar approach for the barplots in Figures 2, 3, 4, and Supplementary Figures 15–17. We again filtered out the low priority associations and selected, for each body site represented in the figure (e.g., oral, skin, nasal) the top N with the greatest difference in absolute value of average L2FC relative to the mid-flight timepoints. In other words, we selected for microbial features with dramatic overall L2FCs. We maximized N based on the available space in the Figure in question. We note that the complete, categorized association results are available in Supplementary Table 12 and in the online data resource, and in creating the figures we did not identify a deviation between the strongest findings there and those presented visually in the text.

### Detecting strain sharing between the crew and environment before, during, and after flight

We modeled our strain-sharing analysis based on Valles-Collomer et al., 2021. Briefly, we used the –s flag in MetaPhlAn4 to generate sam files that could be fed into StrainPhlAn. We used the sample2markers.py script to generate consensus markers and extracted markers for each identified strain using extract_markers.py. We ran StrainPhlAn with the settings recommended by Valles-Collome et al. (--markers_in_n_samples 1, –samples_with_n_markers 10 –mutation_rates –phylophlan_mode accurate). We then used the tree distance files generated by StrainPhlAn to identify strain-sharing cutoffs based on the prevalence of different strains (detailed tutorial: https://github.com/biobakery/MetaPhlAn/wiki/Strain-Sharing-Inference).

### Association with host immune gene subtypes

The single cell sequencing approach and averaging of host genes to identify expression levels is documented in Overbey et al [in review] and Kim at al [in review]. The resultant averaged expression levels across cell types were associated with microbial feature abundance/expression using lasso regression. We used the same log transformation approach as in the mixed effects modeling for the microbial features, and we centered and rescaled the immune expression data. In total, we computed one regression per immune cell type (N = 8) per relevant microbial feature, with the independent variables being all human genes (N = 30,601). We selected features based on their grouping described above, picking only those that were increased transiently or persistently increased after flight. Due to the volume of gene-catalog associations, we only analyzed persistently increased genes. We report outcomes with non-zero coefficients in the text..

### Figure generation and additional data processing notes

The GNU parallel package was used for multiprocessing on the Linux command line.^69^ We additionally used a series of separate R packages for analysis and visualization.^67,68,70–75^ Figures were compiled in Adobe Illustrator.
